# Reference Gene Selection for Quantitative Real-Time RT-PCR Normalization in *Iris. lactea* var. *chinensis* Roots under Cadmium, Lead, and Salt Stress Conditions

**DOI:** 10.1155/2014/532713

**Published:** 2014-05-26

**Authors:** Chun-Sun Gu, Liang-qin Liu, Chen Xu, Yan-hai Zhao, Xu-dong Zhu, Su-Zhen Huang

**Affiliations:** ^1^Institute of Botany, Jiangsu Province and Chinese Academy of Science, Nanjing, Jiangsu 210014, China; ^2^College of Horticulture, Nanjing Agricultural University, Nanjing 210014, China; ^3^College of Landscape Architecture Profiles, Nanjing Forestry University, Nanjing 210014, China

## Abstract

Quantitative real time PCR (RT-qPCR) has emerged as an accurate and sensitive method to measure the gene expression. However, obtaining reliable result depends on the selection of reference genes which normalize differences among samples. In this study, we assessed the expression stability of seven reference genes, namely, ubiquitin-protein ligase UBC9 (*UBC*), tubulin alpha-5 (*TUBLIN*), eukaryotic translation initiation factor (*EIF-5A*), translation elongation factor EF1A (*EF1**α***), translation elongation factor EF1B (*EF1b*), actin11 (*ACTIN*), and histone H3 (*HIS*), in *Iris. lactea* var. *chinensis* (*I. lactea* var. *chinensis*) root when the plants were subjected to cadmium (Cd), lead (Pb), and salt stress conditions. All seven reference genes showed a relatively wide range of threshold cycles (*C*_*t*_) values in different samples. GeNorm and NormFinder algorithms were used to assess the suitable reference genes. The results from the two software units showed that *EIF-5A* and *UBC* were the most stable reference genes across all of the tested samples, while *TUBLIN* was unsuitable as internal controls. *I. lactea* var. *chinensis* is tolerant to Cd, Pb, and salt. Our results will benefit future research on gene expression in response to the three abiotic stresses.

## 1. Introduction

Quantitative real time PCR (RT-qPCR) is a powerful technique to evaluate the quantification of target gene expression. It has advantages of high sensitivity, outstanding accuracy, and broad dynamic range compared with Northern blotting and reverse transcription PCR (RT-PCR) [1]. Nevertheless, it is necessary to use reliable reference gene(s) to normalize the relative expression of target genes. However, the expression of reference gene(s) was not stable under many conditions, which may lead to erroneous normalization [2–4]. As far as is known, many studies have been carried out to select stable reference genes in plants [5, 6].

Cd and Pb are two important heavy metal pollutants which have high toxicity to living beings [7, 8], and salinity is one of the major abiotic stresses which limit the yield of major crops [9]. A number of attempts for reference gene validation have been reported under heavy metal stress in* Arabidopsis thaliana *[10], soybean [11], cucumber [12], citus [2], and poplar [13] and under salt stress in potato [4], rice [14], tobacco [15], cucumber [16], and* Brachypodium distachyon* [17].* I. lactea *var.* chinensis *is a perennial ornamental plant, having potential application in phytoremediation of Cd and Pb [7, 18]. Moreover it is a promising halophyte for the improvement of saline land [9]. To further elucidate the excellent characteristic, more studies are needed to analyze the expression of functional genes and transcription factors under these three abiotic stresses. However, previously studies showed that no single reference gene can be used under various experiment stresses [4, 19, 20]. Thus, it is necessary to identify a set of stable reference genes in* I. lactea *var.* chinensis *under these three stress conditions.

In this study, we used RT-qPCR to examine expression variations of seven candidate reference genes. Then, we compared their stabilities across a large set of* I. lactea *var.* chinensis *samples representing Cd, Pb, and salt stress treatments using GeNorm and NormFinder software units. This work will benefit future gene expression analysis in* I. lactea *var.* chinensis.*

## 2. Materials and Methods

### 2.1. Plant Materials and Treatments

10 cm height* I. lactea* var.* chinensis* plants grown in the 1/2 Hoagland nutrient solution at* Iris* Resource Collection Garden of Institute of Botany, Nanjing Sun Yat-Sen Memorial Botanical Garden, were selected and transferred into 500 mL plastic pots for hydroponic cultivation [7]. After two weeks, the uniform and healthy seedings were used to examine the gene expression patterns under different treatments. For Cd, Pb, or salt treatment, the plants were transferred to pots containing nutrient solution added with 80 mg/L CdCl_2_ [18], or 10 mM Pb(NO_3_)_2_ [7], or 100 mM NaCl stress [9], kept in the same growth pots for designated time (0, 1, 3, 6, 12, and 24 h). The roots were harvested after three treatments. After harvesting, the roots were frozen immediately in liquid nitrogen and stored at −80°C until use for RNA extraction.

### 2.2. RNA Extraction and cDNA Synthesis

Frozen roots were ground in liquid nitrogen using a mortar and a pestle. Total RNA was extracted using the RNAiso reagent (TaKaRa) according to the manufacturer's instructions. Potentially contaminating DNA was eliminated from total RNA with RNase-free DNaseI (TaKaRa). Only RNA samples with an optical density absorption ratio A260/A280 of 1.8–2.0 and an A260/A230 ratio >2.0 were used for subsequent analysis [21]. RNA purity was assessed on a BioPhotometer D30 (Eppendorf) [22]. First-strand cDNA was synthesized with the M-MLV (RNase H^−^) (TaKaRa, Japan) and oligo-dT primers.

### 2.3. Primer Design

The first important step in RT-qPCR reference gene selection is to select an initial set of candidate reference genes. Seven genes that were commonly used as stable reference genes in abiotic stresses were chosen [2, 19, 23] (Table 1). Primers were designed using Primer Premier v5.0 software (Premier Biosoft International) with melting temperatures (*T*_*m*_) of 83.3–90.5°C, primer lengths of 20–22 bp, and amplicon lengths of approximately 110–224 bp (Table 2). Specificity of the amplification product was tested by qPCR. The expected size of the primer amplicons was further verified by agarose gel electrophoresis. Amplicon purity was assumed where a single melting peak was produced.

### 2.4. RT-qPCR

The RT-qPCR reactions were run on a Mastercycler ep* realplex* real-time PCR system (Eppendorf, http://www.eppendorf.com/) with SYBR Premix* Ex Taq* II (Perfect Real Time) (TAKARA). Each reaction was performed in 20 *μ*L mix containing 50 ng of each cDNA, 200 nM of each primer, and 10 *μ*L SYBR Premix. The following amplification program was used: initial denaturation 95°C for 120 s, then 40 cycles of 95°C at 15 seconds, 55°C at 15 seconds, and 72°C at 20 seconds. Melting curves were recorded after cycle 40 by heating from 60 to 95°C at a rate of 0.5°C s^−1^. Each RT-qPCR was run in triplicate, and mean *C*_*t*_ values were calculated. Reverse transcription negative control was also included for each primer pair.

### 2.5. Statistical Analysis

PCR efficiency was calculated from amplification plots using the LinRegPCR program [24]. The quantification cycle values were converted into relative quantities via the delta-Cq method [25]. Two statistical approaches were used to determine the stability of the candidate samples: GeNorm software [26] and NormFinder software[27].

## 3. Results

### 3.1. Performance of the Primers and *C*_*t*_ Value Analysis

A total of seven genes, including* UBC, TUBLIN*,* EIF-5A, EF1*α*, EF1b, ACTIN,* and* HIS, *were selected as reference gene candidates. A single band in gel electrophoresis and a single peak in melt curve indicated the expected amplicons. As shown in Table 2, the correlation coefficients (*R* ^2^) ranged in value between 0.9958 and 0.9997, and PCR amplification efficiencies between 1.905 and 2.016. The two results were from the LinRegPCR program [24].

**Figure 1:**
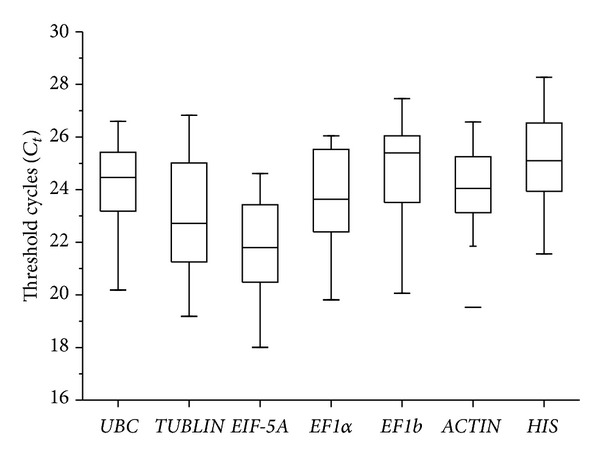
Absolute cycle threshold values (*C*_*t*_) for seven reference genes. Boxes indicate the 25th/75th percentiles, the line marks the median, squares represent the means, and whiskers indicate the ranges for total samples.

In our study, *C*_*t*_ values of the seven reference genes showed a relatively wide range from 18.01 to 28.67 in tested samples (Figure 1). The least abundant transcripts were* HIS* and* EF1b* with *C*_*t*_ values of 28.67 and 27.46, respectively. However,* EIF-5A* presented the highest transcriptional level and the lowest *C*_*t*_ value of 18.01. The average *C*_*t*_ value of the selected genes was about 23.82. The coefficient of variation of* EF1*α** was smallest (6.24), while the coefficient of variation of* TUBLIN *was the largest (7.64).

### 3.2. The Stability of Reference Genes

**Figure 2:**
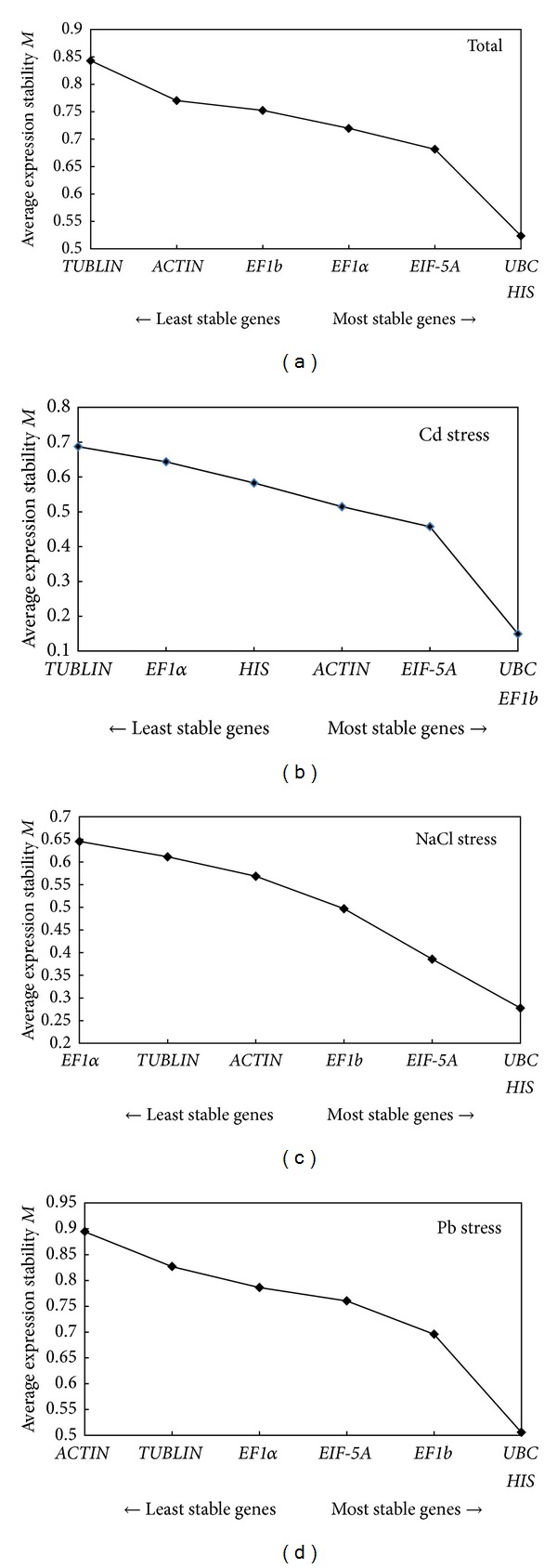
Average expression stability values of control genes by GeNorm analysis: (a) all stresses combined; (b) Cd stress exposure; (c) NaCl stress exposure; (d) Pb sress exposure. The least stable genes are on the left, and the most stable on the right.

**Figure 3:**
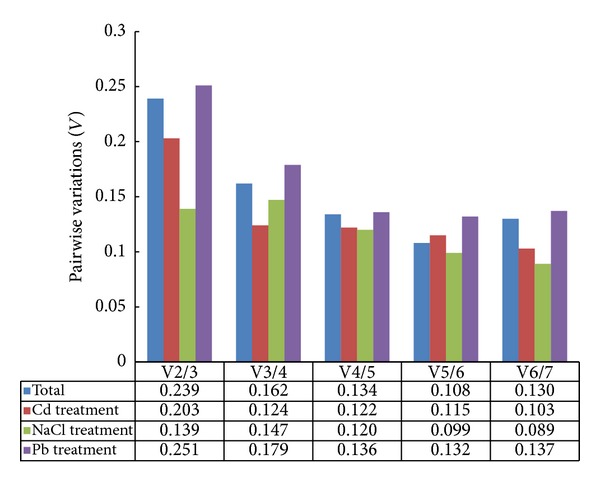
Determination of the optimal number of reference genes by GeNorm analysis.

In our study, two methods were selected to analyze the stability of seven reference genes. GeNorm calculates *M* (average expression stability) for the identification of the most suitable reference gene(s) and *V* (average pairwise variation) to define the optimal number of genes that should be used. On the basis of *M*, a lower *M* value indicates more stable genes. Genes which had *M* values more than 1.5 indicated the need for additional reference gene(s) [22]. The ranking order according to the *M* value was showed in Figure 2. The *M* values for all genes were below 1.5. For total samples,* UBC* and* HIS* were the most stably expressed genes with an *M* value of 0.524.* ACTIN* and* TUBLIN* were the least stable genes (Figure 2(a)). In different samples across Cd treatment,* UBC* and* EF1b* performed well with an *M* value of 0.149, while* EF1*α** and* TUBLIN* have relative high *M* value (Figure 2(b)). For NaCl treatment,* UBC* and* HIS* were the most highly ranked with an *M* value of 0.278.* EF1*α** and* TUBLIN* were the least stable genes like under NaCl treatment (Figure 2(c)). For Pb treatment,* UBC *and* HIS* showed the lowest *M* value of 0.506 and* ACTIN* was the highest with an *M* value of 0.894 (Figure 2(d)). Vandesompele defined *V* (the pairwise variation *V*_*n*_/*V*_*n*+1_) to choose the optimal number of reference genes [26]. As showed in Figure 3, three groups of samples, that is, total samples, Cd stress treatment samples, and Pb stress treatment samples, showed higher V2/3 value more than 0.15 (Figure 3). Thus, three reference genes in Cd stress and four reference genes in total and Pb stress were necessary to obtain accurate results in gene expression normalization.

GeNorm and NormFinder were developed based on a different strategy. Each one has its own advantages and disadvantages [19]. To further confirm the result obtained by the GeNorm software, we further analyzed using NormFinder software, an algorithm which depends on a statistical and mathematical model that estimates the overall expression variation of a set of candidates to identify the optimal normalization gene [21, 27]. Results indicated that the most unstable gene in total, Cd stress, NaCl stress, and Pb stress, was consistent with the GeNorm analysis (Table 3).* EIF-5A *ranked as the most stable gene in total (stability value = 0.295) and Cd stress (stability value = 0.084).* His* was optimal with a stability value of 0.194 the NaCl treatment. During the Pb stress,* UBC* ranked in the top in NormFinder analysis.

## 4. Discussion

Many studies have been performed to evaluate and select reliable reference genes in a number of plants, such as in cotton subjected to salt and drought stress [19], in* Lycium barbarum* L. under different development stages [28], in* chrysanthemum* during aphid infestation, heat stress, or waterlogging stress [22], in spathe tissue of* Anthurium andraeanum *[29], and in* Coffea arabica* during nitrogen starvation, salt, and heat stress [30]. Here, we have found that seven candidate reference genes performed differently upon three stresses to which* I. lactea *var.* chinensis* plants were subjected.

Two commonly used algorithms (GeNorm and NormFinder) were used to evaluate and identify reference genes. The GeNorm analysis may be biased by the coregulation, since they will show a lower level of pairwise variation than independently regulated genes and occupy closed positions in the ranking coregulated genes [15, 26]. However, NormFinder could be more effective in avoiding behavior of gene coregulation because it ranks reference genes according to the intra- and intergroup variation [31, 32]. Our study showed that different gene stability ranking orders were generated by two analysis algorithms. In previous studies, different conclusions were also generated by the two methods such as in* Cineraria* [33],* C. lavandulifolium* [34], radish [32], cucumber [16], and flax [35]. Based on our study,* EIF-5A *and* UBC *exhibited stable expression patterns for accurate normalization when looking at the expression data in all four series. On the other hand,* TUBLIN* performed poorly which indicated that it was not consistently expressed and should not be used as reference genes in our experimental setups. In addition, GeNorm results showed that the choice of the reference gene number depends on the experiment conditions (Figure 3).

*EIF-5A* and* UBC* were abundantly and constantly transcribed in all of the samples. Indeed, EIF-5A is thought to function in protein synthesis by promoting synthesis of the first peptide bond [36]. UBC is known to be ubiquitin conjugating enzyme [37]. So they remained continuously expressed over the different conditions and showed minimal changes in RNA transcription. The most commonly used reference gene,* ACTIN*, was not among the more stable genes in our tests. Previously,* ACTIN* was commonly used as endogenous internal controls to normalize gene expression studies [38]. However, the poor stability of* ACTIN* was found in potato [4], peach [31], and in cucumber [16]. It may be that the total actin content can vary with development, cell culture conditions, and potentially between cells within tissues [38].* EF1*α** and* EF1b* belong to elongation factor-1 gene family. However,* EF1*α** was ranked above* EF1b *except for NaCl and Pb stress (Table 3). They were not among the best reference genes in our test just as the earlier analyses [10, 32]. This may be that the expression of* EF1 *can be modulated in situations involving growth restriction, transformation, ageing, and cell death [39]. Compared with* EF1*α** and* EF1b, TUBLIN *was the least stably expressed gene found. Surprisingly,* TUBLIN* showed highly stable expression in longan tree [40] and in cucumber [16].* HIS *was the most stably expressed gene under NaCl stress, but it was not suitable as the best under three analyses (Table 3). These results suggest that we should choose suitable reference genes according to different species and conditions.

## 5. Conclusion

To our knowledge, this study is the first systematic analysis for the selection of superior reference genes for qPCR in* I. lactea *var.* chinensis* roots under different abiotic (Cd, NaCl, and Pb) stress conditions. Analysis using GeNorm and NormFinder algorithms revealed that* EIF-5A* and* UBC* could be considered to be appropriate reference genes for gene expression analysis under different abiotic experiment stress, whereas* TUBLIN* showed relatively low expression stability. This work will enable accurate and reliable gene expression experiments under different abiotic stress conditions in* I. lactea *var.* chinensis *root.

## Figures and Tables

**Table 1:** Description of *Iris. lactea *var.* chinensis* reference genes for RT-qPCR.

Gene^a^	NCBI accession number	Arabidopsis ortholog locus^b^	Arabidopsis locus description
*UBC *	EX953716	AT4g27960	Ubiquitin-protein ligase UBC9
*TUBLIN *	EX954248	AT5G19780	Tubulin alpha-5
*EIF-5A *	EX954588	AT1G69410	Eukaryotic translation initiation factor
*EF1*α**	EX950257	AT5G60390	Translation elongation factor EF1A
*EF1b *	AB907790	AT2G18110	Translation elongation factor EF1B
*ACTIN *	EX952640	AT3G12110	ACT11
*HIS *	FD387291	AT4G40040	Histone H3

^a^All genes were named on the basis of similarity to Arabidopsis proteins determined via BLASTX.

^b^Closest Arabidopsis homolog identified using TAIR BLAST (http://www.arabidopsis.org/Blast/index.jsp).

**Table 2:** Primer sequences and amplicon characteristics for each of the seven reference genes.

Name	Primer sequence (forward/reverse primer)	Size (bp)	*T*_*m*_ (°C)	*E* (%)	*R* ^2^
*UBC *	5′-TCTCGCTTGTCCGGTTTGTG-3′	224	88.0	1.965	0.9993
	5′-ACCTTGGGTGGCTTGAATGG-3′				
*TUBLIN *	5′-TTACCGTCAACTATTCCACCCA-3′	214	87	1.905	0.9991
	5′-CAGCAACGAACCCAAACCAGAT-3′				
*EIF-5A *	5′-GGATGAGGAGCACCACTTCG-3′	110	90.5	1.912	0.9997
	5′-GGCGGTTCTTGATGACGATG-3′				
*EF1*α**	5′-CCATTTCTGGATTTGAGGGTGA-3′	133	86.5	2.016	0.9958
	5′-AGTCGAAGAGGCTTGTCGGTAG-3′				
*EF1b *	5′-ATCTTCTGACCAGGAGTTACAT-3′	115	83.3	2.006	0.9997
	5′-TACCACCTAGCAACATTGAC-3′				
*ACTIN *	5′-CTCAACCCGAAGGCAAACAGAG-3′	216	87.0	1.966	0.9994
	5′-CGCAAGGTCCAGACGGAGAATA-3′				
*HIS *	5′-GGCTCGTACCAAGCAAACTG-3′	134	89.4	1.929	0.9993
	5′-TTCCAGGACGGTAACGATGA-3′				

**Table 3:** Ranking of seven reference genes in order of their expression stability calculated by NormFinder.

Ranking order	Total		Cd		NaCl		Pb	
	Gene	Stability	Gene	Stability	Gene	Stability	Gene	Stability
1	*EIF-5A *	0.295	*EIF-5A *	0.084	*HIS *	0.194	*UBC *	0.263
2	*UBC *	0.308	*ACTIN *	0.296	*UBC *	0.225	*EIF-5A *	0.332
3	*EF1*α**	0.374	*UBC *	0.310	*ACTIN *	0.308	*EF1b *	0.364
4	*EF1b *	0.379	*EF1*α**	0.366	*EIF-5A *	0.342	*HIS *	0.396
5	*ACTIN *	0.448	*EF1b *	0.367	*TUBLIN *	0.367	*EF1*α**	0.446
6	*HIS *	0.450	*HIS *	0.442	*EF1b *	0.371	*TUBLIN *	0.599
7	*TUBLIN *	0.613	*TUBLIN *	0.480	*EF1*α**	0.411	*ACTIN *	0.643
